# Internal carotid bulb occlusion by a giant thrombus after thoracoscopic left upper lung lobectomy successfully treated with endovascular stenting: a case report

**DOI:** 10.1186/s44215-023-00116-4

**Published:** 2023-11-28

**Authors:** Tomohito Saito, Takenobu Kunieda, Yasumasa Hashimoto, Mitsuaki Ishida, Natsumi Maru, Takahiro Utsumi, Hiroshi Matsui, Yohei Taniguchi, Haruaki Hino, Tomohiro Murakawa

**Affiliations:** 1 Department of Thoracic Surgery, Kansai Medical University, Hirakata, Osaka, 573-1010 Japan; 2 Department of Neurology, Kansai Medical University, Hirakata, Osaka, Japan; 3 Department of Molecular Therapy, National Institute of Neuroscience, National Center of Neurology and Psychiatry, Kodaira, Japan; 4 Department of Pathology, Osaka Medical and Pharmaceutical University, Osaka, Japan

**Keywords:** Acute ischemic stroke, Carotid occlusion, Endovascular stenting, Endovascular thrombectomy, Hypercoagulable state, Left upper lung lobectomy

## Abstract

**Background:**

Perioperative acute ischemic stroke following lung resection is relatively rare, though a devastating complication. Specifically, patients undergoing left upper lung lobectomy for lung cancer are likely to develop perioperative acute ischemic stroke.

**Case presentation:**

A 67-year-old man underwent thoracoscopic left upper lung lobectomy for lung adenocarcinoma; he experienced sudden-onset left hemiparesis and dysarthria on the morning of the second postoperative day. Angiography revealed occlusion of the bulbs of the right internal and external carotid arteries by a giant thrombus, which could not be removed through endovascular thrombectomy. We deployed a stent at the right carotid bifurcation to foist the giant thrombus, achieving revascularization 4 h after the onset. Treatment response was assessed as good improvement with a modified Rankin scale score of 0, and the patient was discharged home 19 days after symptom onset.

**Conclusions:**

We present a unique case of carotid bulb thromboembolism resulting from a giant thrombus following thoracoscopic left upper lung lobectomy, for which endovascular stenting was effective.

## Background

Perioperative acute ischemic stroke (AIS) is a relatively rare (approximately 0.6%) but devastating complication of lung resection that results in an adjusted 13-fold increase in the inhospital mortality [1–3]. Specifically, patients undergoing left upper lung lobectomy (LUL) for lung cancer are likely to develop perioperative AIS (incidence: 1.5–4.5%) [2, 4]. We herein report a case of internal carotid bulb occlusion by a giant thrombus following LUL that was successfully treated with endovascular stenting.

## Case presentation

**Fig. 1:**
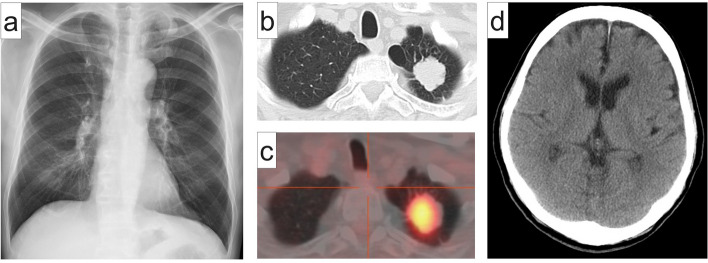
Preoperative imaging. **a** Chest radiograph shows an abnormal shadow in the left upper lung field. **b** Chest computed tomography image reveals a 31-mm solid nodule in the left upper lung lobe (S1+2). **c** Increased 18F-fluorodeoxyglucose uptake is seen within the nodule in the left upper lung lobe. **d** Brain contrast-enhanced computed tomography image reveals no sign of brain metastasis

A 67-year-old man with a 40 pack-year smoking history and a medical history of hyperuricemia and hypertension presented with an abnormal shadow in his left upper lung field (Fig. 1a). Chest computed tomography (CT) revealed a 31-mm solid nodule in the left upper lung lobe (S1+2), which showed increased 18F-fluorodeoxyglucose uptake (Fig. 1b and c). Brain contrast-enhanced CT showed no signs of brain metastasis (Fig. 1d). Thus, surgery was scheduled for suspected lung cancer (cT2aN0M0 disease). The serum carcinoembryonic antigen level was elevated to 9.5 ng/mL.

**Fig. 2:**
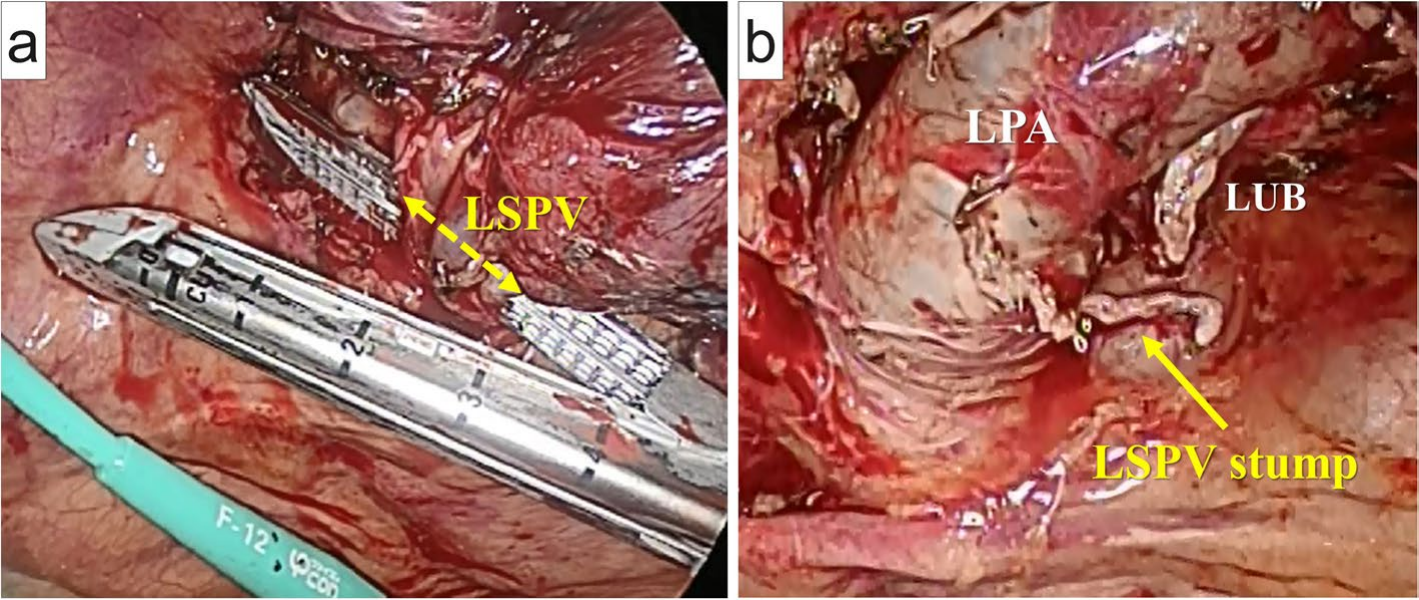
Thoracoscopic imaging. **a** The left superior pulmonary vein (LSPV), which did not form a common trunk with the left inferior pulmonary vein, is divided using an endoscopic linear stapler following division of the left pulmonary artery (LPA) branches and prior to division of the left upper bronchus (LUB). **b** The stump of the LSPV is seen. LPA, left pulmonary artery; LSPV, left superior pulmonary vein; LUB, left upper bronchus

Intraoperative frozen section analysis revealed an adenocarcinoma; thus, the patient underwent LUL with lobe-specific nodal dissection via thoracoscopy. Pulmonary artery branches were stapled and initially divided during the surgery. The left superior pulmonary vein (PV) was divided by an endoscopic linear stapler (Fig. 2a and b). Finally, the left upper bronchus was stapled and divided. The final pathological diagnosis was solid-predominant adenocarcinoma (pT2aN0M0, pStage IB). Intraoperative electrocardiography revealed a normal sinus rhythm throughout the surgery.

**Fig. 3:**
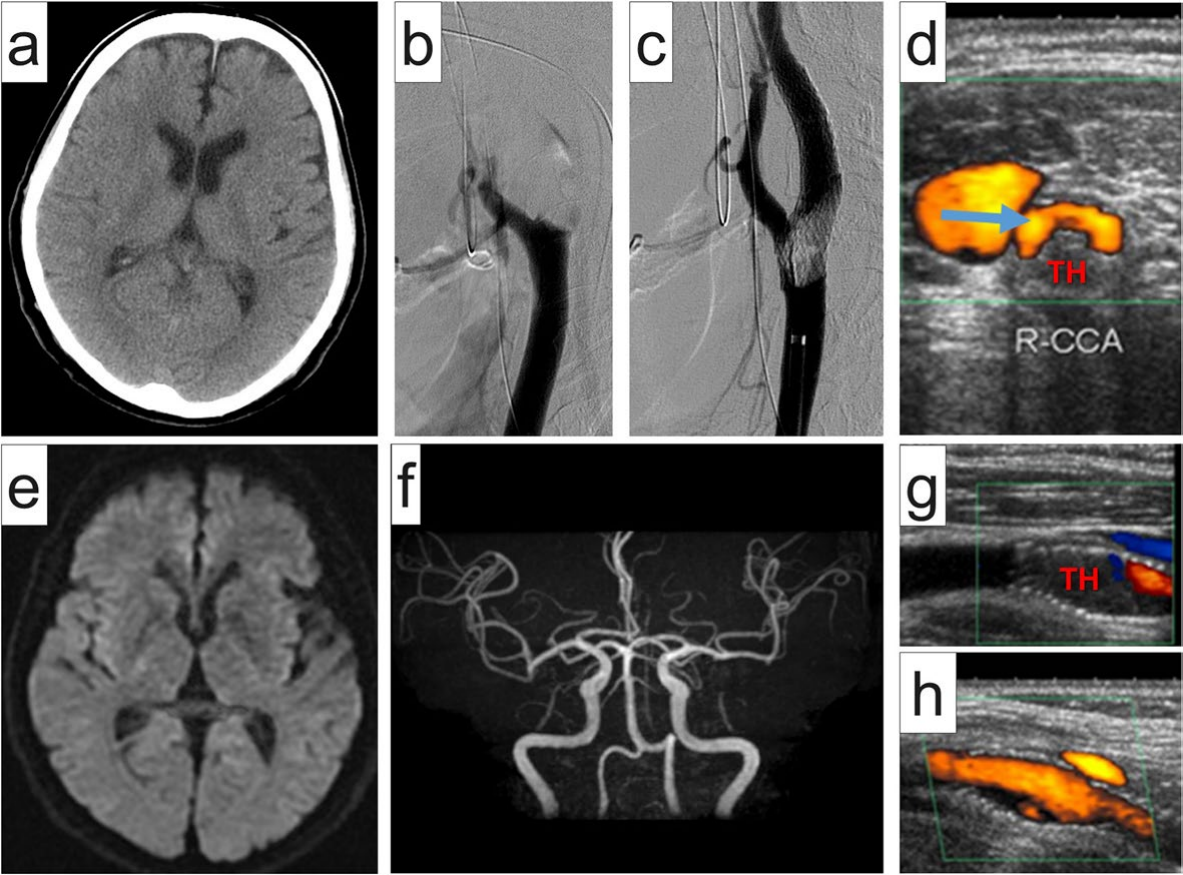
Imaging of the occluded right internal and external carotid arteries treated with endovascular stenting. **a** Head computed tomography image reveals neither intracranial hemorrhage nor early signs of brain infarction at the onset. **b** Angiography image shows that the bulb of the right internal carotid artery and the right external carotid artery is occluded by a giant thrombus. **c** The right internal and external carotid arteries are successfully revascularized by introducing a 10 mm × 40 mm PRECISE stent (Cordis, Miami, FL, USA) at the right carotid bifurcation. **d** Carotid Doppler ultrasonography image shows that the giant thrombus (TH) remains within the right common carotid artery, protruding from the stent. The blue arrow indicates the recanalized lumen. **e** Magnetic resonance image obtained on the day after recanalization shows no sign of cerebral infarction. **f** Magnetic resonance angiography image shows the patency of the vasculature (including the right internal carotid artery). **g** Carotid Doppler ultrasonography image shows that the thrombus (TH) remains within the right internal carotid artery 10 days after recanalization. **h** Carotid Doppler ultrasonography image shows that the thrombus has completely disappeared 67 days following recanalization

On the morning of the second postoperative day, the patient suddenly developed left hemiparesis and dysarthria with the following vital signs: blood pressure, 130/69 mmHg; heart rate, 60/min; and respiratory rate, 18/min. Twelve-lead electrocardiography revealed a normal sinus rhythm. The D-dimer level was 0.9 μg/mL (reference: ≤ 1.0 μg/mL). “Code stroke” was activated, and the initial neurological examination revealed National Institute of Health Stroke Scale score of 15. Head CT revealed neither signs of intracranial hemorrhage nor early signs of cerebral infarction (Fig. 3a). Emergency carotid endovascular revascularization was initiated at 2 h 50 min following symptom onset. Contrast-enhanced chest CT was not conducted at this time because we prioritized the revascularization. The patient did not consent to transesophageal echocardiography; thus, transthoracic echocardiography was performed, but no signs of a PV stump thrombus or an intracardiac shunt were noted.

An angiogram revealed that the bulb of the right internal carotid artery (ICA) and the right external carotid artery was occluded by a giant thrombus (Fig. 3b). We attempted an endovascular forced-suction thrombectomy but failed to remove the giant thrombus. Thus, we decided to deploy a 10 mm × 40 mm PRECISE stent (Cordis, Miami, FL, USA) at the right carotid bifurcation to foist the giant thrombus, which finally lead successful revascularization (Fig. 3c) at 4 h 22 min after the onset. Carotid Doppler ultrasonography demonstrated that the giant thrombus remained in the right common carotid artery, protruding from the stent (Fig. 3d). Immediately following recanalization, the patient’s symptoms improved; the National Institute of Health Stroke Scale score dropped to 1 and 0 at 1 and 3 h after revascularization, respectively. Antithrombotic therapy with clopidogrel (75 mg/day) and aspirin (200 mg/day) was initiated; this was switched to warfarin and clopidogrel (75 mg/day) 7 days after revascularization. The day after revascularization, magnetic resonance imaging revealed no signs of cerebral infarction (Fig. 3e) or carotid artery occlusion (Fig. 3f). Additionally, chest contrast-enhanced CT revealed no signs of PV stump thrombosis. Holter electrocardiography revealed no evidence of atrial fibrillation. Carotid Doppler ultrasonography showed that the thromboemboli still remained within the right ICA 10 days after recanalization (Fig. 3g) but disappeared completely 67 days after recanalization (Fig. 3h). The patient’s condition had improved by a good degree (modified Rankin scale score: 0); the patient was discharged home 19 days after symptom onset. No neurological sequelae were noted for 5 years thereafter.

## Discussion

A limitation in our case was that we could not identify the source of thromboembolism. Most cases of perioperative AIS are attributed to large-artery atherosclerosis and cardioembolism; however, one-fourth of the cases remain cryptogenic following standard diagnostic evaluation [5]. Cryptogenic AIS that was deemed nonlacunar and nonatherosclerotic is characterized as an embolic stroke of undetermined source (ESUS) [6], accounting for approximately 17% of all AIS cases [7]. Emerging evidence suggests that a cancer-mediated hypercoagulability may cause ESUS [8–10]; currently, reducing cancer activity, including by surgical resection, seems the only way to attenuate the cancer-mediated hypercoagulability and the subsequent risk of AIS development [8].

Based on the current prevailing hypothesis, perioperative AIS following LUL for lung cancer may result from floating thromboemboli that develop due to a turbulent blood flow within a long PV stump [11–14]. To date, two preventive strategies have been proposed to avoid AIS after a pulmonary lobectomy [15]. The first strategy is the creation of a short PV stump; some surgeons have proposed PV ligation at the level of the pericardial reflection [16, 17]. In light of our case, we started to apply a “dissecting PV last” approach to shorten the PV stump [18]. However, cardiac tamponade may develop if the staple line of the PV involves the pericardial reflection [19, 20]. The second strategy is the early detection of PV stump thrombosis. Contrast-enhanced CT and transesophageal echocardiography are suggested imaging modalities for the detection of PV stump thrombosis; contrast-enhanced CT seems preferrable in post-lobectomy settings for its feasibility [12]. Given that postoperative AIS is likely to develop within 1 week from pulmonary resection [4, 13, 21], routine contrast-enhanced CT is recommended within 1 week postoperatively [15]. If PV stump thrombosis is detected, anticoagulation therapy should be considered in accordance with the management of left atrial thrombosis [22]. In fact, the use of intravenous heparin followed by oral warfarin reportedly leads to the dissipation of PV stump thrombus [23, 24]. Further research is necessary to identify the optimal anticoagulation strategy in balance with the risk of bleeding.

Undoubtedly, appropriate and prompt diagnostic and therapeutic interventions in patients with suspected perioperative AIS are critical for improving their prognosis. “Code stroke,” a rapid response system that prioritizes the hyperacute assessment and management of patients with stroke, is key to optimizing inhospital stroke care [25]. Our hospital introduced a code stroke system in 2010. Our code stroke response team comprises two neurologists and one neuro-interventionist, who are prepared to take action 24/7. According to the 2020 guidelines from the Society for Neuroscience in Anesthesiology and Critical Care, recombinant tissue plasminogen activator is relatively contraindicated following a major surgery within 14 days, while immediate mechanical thrombectomy is recommended for patients with large-vessel occlusion if all selection criteria are met [5]. Thus, endovascular thrombectomy might be a good treatment choice for AIS with large-vessel occlusion following lung resection.

To the best of our knowledge, only 12 cases (including the current case) of endovascular treatment of perioperative AIS following lung resection (Table 1) have been presented to date [26–33]. Our case appears to be the first on endovascular stenting following failed endovascular thrombectomy in a patient who developed perioperative AIS after lung resection. Among the 12 patients, six (50.0%) were finally discharged home, five (41.7%) were transferred to another hospital, and one (8.3%) died of cerebral herniation. Notably, endovascular thrombectomy failed to achieve recanalization in three of the 12 (25.0%) patients. These outcomes are comparable to those reported in a recent meta-analysis on endovascular thrombectomy (mortality, approximately 15%; failure to achieve revascularization, approximately 29%) [34].

**Table 1:** Summary of reported cases and clinical outcomes of perioperative acute ischemic stroke following lung resection treated by endovascular treatment

Authors, year	Age, sex	Surgery type	Onset	PVT	Occluded vessel	Symptom	EVT type	Clinical outcome
Ikeda et al. (2014) [26]	58, M	LUL	Day 2	Yes	Lt. ICA	Hemiplegia, aphasia	Thrombectomy	Discharged home 27 days after the onset with “hemiplegia and aphasia”
Sonobe et al. (2019) [27]	67, M	LP	Day 11	Yes	Rt. MCA	Hemiparesis	Thrombectomy	Discharged home 49 days after the onset with an mRS of 1
Kimura et al. (2019) [28]	76, M	LUL	Day 2	No	Rt. MCA	Hemiplegia	Thrombectomy	Discharged home (time unknown) with “full recovery”
	67, F	LLL	Day 4	No	Lt. MCA	Aphasia, somnolence	Thrombectomy	Discharged home (time unknown) with “mild aphasia”
Usui et al. (2019) [29]	68, M	LLL	Day 1	No	Lt. MCA	Hemiplegia	Thrombectomy	Discharged home (time unknown) with an NIHSS of 0
Masahira et al. (2020) [30]	61, M	LLL	Day 1	No	Rt. MCA	Hemiplegia	Thrombectomy	Transferred to another hospital 13 days after the onset with an mRS of 1
	68, M	RLL	Day 0^a^	No	Rt. MCA	Hemiplegia	Thrombectomy	Transferred to another hospital 46 days after the onset with an mRS of 3
Morinaga et al. (2019) [31]	71, M	LUL	Day 0	N/A	Lt. MCA	N/A	Thrombectomy	Transferred to another hospital (time unknown) with an mRS of 3
	76, M	LLL	Day 6	N/A	Lt. MCA	N/A	Thrombectomy	Transferred to another hospital (time unknown) with an mRS of 1
Kishida et al. (2013) [32]	75, M	LUL	Day 50	Yes	Lt ICA	Hemiplegia, aphasia	Thrombectomy^b^	Transferred to another hospital (time unknown) with an mRS of 5
Fujii et al. (2020) [33]	71, F	LUDS	Day 4	N/A	Rt. ICA	Hemiplegia	Thrombectomy^c^	Died of cerebral herniation 9 days after the onset
Present case	67, M	LUL	Day 2	No	Rt. ICA	Hemiplegia	Stenting#	Discharged home 19 days after the onset with an mRS of 0

*EVT*, endovascular treatment; *ICA*, internal carotid artery; *LP*, left pneumonectomy; *LUDS*, left upper division segmentectomy; *LUL*, left upper lung lobectomy; *MCA*, middle cerebral artery; *mRS*, modified Rankin Sale; *NIHSS*, the National Institute of Health Stroke Scale; *PVT*, pulmonary vein thrombosis; *RLL*, right lower lung lobectomy

^a^Three hours after the operation. ^b^Thrombectomy was unable to achieve recanalization. ^c^ICA was recanalized, but the distal vessels (M1 segment of the MCA) were subsequently occluded

In the present case, the thrombus’s size may be the main cause of the failed thrombectomy. Carotid arteries occluded due to giant thrombi are reportedly difficult to revascularize [35, 36]. Diabetes mellitus is a reported independent risk factor for failed mechanical thrombectomy [37]. One study indicated a possible association between the onset-to-groin-puncture time and failed mechanical thrombectomy [37], but another did not [38]. Both atrial fibrillation- and PV stump-associated thrombi are caused due to a turbulent blood flow; however, they majorly differ in terms of the neutrophil and erythrocyte content [39]. In patients with a failed thrombectomy, atrial fibrillation may be associated with recanalization success [38]; however, it remains unclear whether PV stump-based thrombosis are also associated with recanalization success.

Endovascular stenting using self-expandable stents is effective for AIS in the context of carotid circulation [40–44]. Rescue stenting seems to be a valid option for AIS following a failed endovascular thrombectomy [44, 45]. Although rescue stenting requires glycoprotein IIB/IIIa antagonists or oral antiplatelet medications to prevent intra-stent thrombosis, these medications are not associated with symptomatic intracranial hemorrhage [46]. A potential therapeutic alternative for the presented case was catheter crushing and aspiration of the thrombus; however, this could have resulted in distal embolism [36]. Thus, rescue endovascular stenting might be a reasonable therapeutic option for post-lobectomy internal carotid bulb occlusion with a giant thrombus refractory to endovascular forced-suction thrombectomy.

In conclusion, we present a unique case of carotid bulb thromboembolism resulting from a giant thrombus following thoracoscopic LUL, for which endovascular stenting was effective. Further studies should establish an optimal treatment strategy for perioperative AIS secondary to a giant thrombus following lung resection.

## Data Availability

The datasets supporting the conclusions of this article are included within the published article.

## Abbreviations

AIS: Acute ischemic stroke
LUL: Left upper lung lobectomy
CT: Computed tomography
PV: Pulmonary vein
ICA: Internal carotid artery
ESUS: Embolic stroke of undetermined source
PVS: Pulmonary vein stenosis
